# Fraudulent News Headline Detection with Attention Mechanism

**DOI:** 10.1155/2021/6679661

**Published:** 2021-03-15

**Authors:** Hankun Liu, Daojing He, Sammy Chan

**Affiliations:** ^1^Software Engineering Institute, East China Normal University, Shanghai 200062, China; ^2^Department of Electrical Engineering, City University of Hong Kong, Hong Kong 999077, China

## Abstract

E-mail systems and online social media platforms are ideal places for news dissemination, but a serious problem is the spread of fraudulent news headlines. The previous method of detecting fraudulent news headlines was mainly laborious manual review. While the total number of news headlines goes as high as 1.48 million, manual review becomes practically infeasible. For news headline text data, attention mechanism has powerful processing capability. In this paper, we propose the models based on LSTM and attention layer, which fit the context of news headlines efficiently and can detect fraudulent news headlines quickly and accurately. Based on multi-head attention mechanism eschewing recurrent unit and reducing sequential computation, we build Mini-Transformer Deep Learning model to further improve the classification performance.

## 1. Introduction

With the rapid development of Internet, Internet security is suffering from various potential threats. The rise of Advanced Persistent Threat (APT) has caused traditional network defense systems to face increasingly severe challenges. According to statistics, social engineering is the main technique that attackers use to launch APT attacks, so it is of practical significance to research on defending against social engineering attacks. Cutting off the chains of attacks, detecting attacks and isolating attackers is the fastest and most effective method of defending against social engineering attacks.

Currently, in major cases of social engineering attacks, the essential operation of attackers to launch attacks is to distribute fraudulent news headlines on e-mail systems and online social media platforms, such as Instant Messaging services (e.g., QQ, WeChat, WhatsApp, Facebook Messenger, and Line) or microblogs (e.g., Twitter and Weibo). Some fraudulent news headlines often carry malicious links preset by attackers. Many curious users who see those news headlines would want to learn more about the detailed contents of those news by clicking directly on the malicious links, which leads to serious consequences, including personal privacy theft, account and password stealing, and even huge asset loss.

According to Symantec Internet Security Threat [1] (ISTR Volume 23), for the social engineering attacks on companies, 71.4% of targeted attacks in 2017 involved the use of spear-phishing e-mails. Therefore, the main vector of social engineering attacks to reach companies through their employees remains e-mail system.

Above all, it is of great importance to analyze and detect fraudulent news headlines, which has a profound impact on Internet security and the defense system against social engineering attacks.

In recent years, Deep Learning models, such as Long Short-Term Memory (LSTM) [2], attention layer [3] and Transformer [4], have demonstrated outstanding advantages in solving the problems of Natural Language Processing (NLP). In this paper, for the classification of news headline text data, we add one extra attention layer to the LSTM model and achieve a slight increase in accuracy. In addition, based on multi-head attention, we build Mini-Transformer without complex recurrent or convolutional neural networks to improve the classification performance (i.e., accuracy, precision, recall, and F1 score) dramatically.

## 2. Related Work

Although a considerable amount of literature has been published on Internet social engineering, the emerging security issues with e-mail systems and online social media platforms are still not addressed adequately. Moreover, since the operational principle of social engineering attacks has not been clearly revealed, it is difficult to construct an effective defense system.

Castillo et al. [5] raised the issue of fake information detection on Twitter. To examine newsworthy topics on Twitter, they evaluated various classification algorithms and analyzed four features (message, user, topic, and propagation). Automatic method was used to classify the credibility of Twitter messages and achieved high precision and recall.

Ma et al. [6] utilised Recurrent Neural Networks (RNN), including LSTM and Gated Recurrent Unit (GRU), to process massive text data. They proposed a novel method that learns continuous representations of microblog events for identifying rumors on Twitter and Weibo more quickly and accurately.

Guo et al. [7] investigated the relevant characteristics of social media and utilised attention mechanism to analyze the massive news and messages on the microblog. They designed an efficient classification scheme, which can detect rumors more accurately.

Song et al. [8] combined LSTM with attention mechanism and proposed a novel method of sentiment lexicon embedding for aspect-level sentiment analysis, which is better at representing sentiment word's semantic relationships to improve the sentiment classification performance.

Vaswani et al. [4] proposed a new network architecture (Transformer) based solely on attention mechanism, which is not only superior in machine translation quality but also more parallelizable so as to require significantly less time to train.

Our work focuses on the news headlines spread on e-mail systems and online social media platforms. We develop a set of models to detect massive fraudulent news headlines using LSTM and attention mechanism. To further improve the classification performance, we build Mini-Transformer, which consists of multi-head attention layers and fully connected dense layers rather than recurrent unit layers (i.e., LSTM layer and GRU layer).

## 3. Methodology

In this section, firstly, we briefly revisit LSTM [2]. Then, we present the formulations of attention layer proposed by Bahdanau et al. [3]. Finally, we show how we use multi-head attention mechanism to build Mini-Transformer.

### 3.1. Long Short-Term Memory (LSTM) Networks

LSTM is able to process variable-length input sequences by recursive operation [2]. With the ability to maintain the hidden states and fit the variations of contextual information in relevant time steps, LSTM is well-suited for classifying news headline text data.

Unlike the traditional Vanilla RNN unit whose hidden state is overwritten in each time step, LSTM unit maintains long memory cell state *c*_*t*_ in time step *t*. Given an input sequence *X* = {*x*_1_, *x*_2_, *x*_3_,…, *x*_len_} with length len, {*x*_*t*_*|*1 ≤ *t* ≤ len} are real number vectors with dimension *d*_*x*_, hidden state sequence {*h*_1_, *h*_2_, *h*_3_,…, *h*_len_} with length len, {*h*_*t*_*|*1 ≤ *t* ≤ len} are real number vectors with dimension *d*_*h*_, and long memory cell state sequence {*c*_1_, *c*_2_, *c*_3_,…, *c*_len_} with length len, {*c*_*t*_*|*1 ≤ *t* ≤ len} are also real number vectors with dimension *d*_*h*_. From *t* = 1 to len, the algorithm iterates as follows:

(1) $$\begin{array}{c} f_{t}\;=\;\sigma \left(W_{f}\cdot \left[\;h_{t-1},x_{t}\;\right]\right),\\ i_{t}\;=\;\sigma \left(W_{i}\cdot \left[\;h_{t-1},x_{t}\;\right]\right),\\ {\overset{˜}{c}}_{t}\;=\;\tanh\left(\;W_{c}\cdot \left[\;h_{t-1},x_{t}\;\right]\;\right),\\ o_{t}\;=\;\sigma \left(W_{o}\cdot \left[\;h_{t-1},x_{t}\;\right]\right),\\ c_{t}\;=\;f_{t}\;*\;c_{t-1}\;+\;i_{t}\;*{\overset{˜}{\;c}}_{t},\\ h_{t}=\;o_{t}\;*\;\tanh\left(c_{t}\right), \end{array}$$

where *W*_*f*_, *W*_*i*_, *W*_*c*_, and *W*_*o*_ are weight matrices for forget gate, input gate, long memory cell, and output gate, respectively. The operator “*·*” denotes the dot-product between the matrix and vector. The operator “*∗*” denotes the element-wise multiplication (Hadamard product) between two vectors. *σ*(·) is the logistic sigmoid function, and tanh(·) is the hyperbolic tangent function.

**Figure 1:**
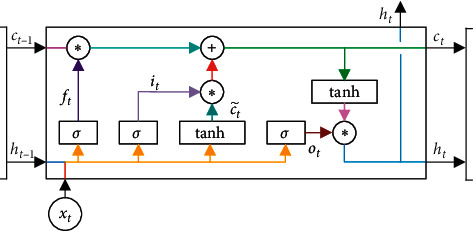
An illustration of LSTM unit.

In LSTM unit, forget gate *f*_*t*_ controls the range of existing memory *c*_*t*−1_ removed from *c*_*t*_, input gate *i*_*t*_ controls the range of new memory ${\tilde{c}}_{t}$ added to *c*_*t*_, and output gate *o*_*t*_ determines the amount of output memory. By removing part of the existing memory *c*_*t*−1_ and adding part of the new memory ${\tilde{c}}_{t}$, long-term memory cell *c*_*t*_ is updated. LSTM unit is illustrated in Figure 1.

From *t* = 1 to len, after all iterative steps of the algorithm, last hidden state vector *h*_len_ is computed to generate real number output *y* via a fully connected dense layer whose activation is logistic sigmoid function.

### 3.2. Attention Layer

In 2014, Bahdanau et al. [3] introduced the attention mechanism to the NLP field for the first time and completed modeling, transduction, and alignment procedure on the machine translation task at the same time.

LSTM layer needs to return all hidden states {*h*_*t*_*|*1 ≤ *t* ≤ len} as the input of attention layer. In attention layer, attention weight scores {*α*_1_, *α*_2_, *α*_3_,…, *α*_len_} are computed with *v*_*α*_, *W*_*α*_, and input sequence {*h*_1_, *h*_2_, *h*_3_,…, *h*_len_}. {*α*_*i*_*|*1 ≤ *i* ≤ len} are real numbers reflecting the importance of each state *h*_*i*_. As trainable parameters, *v*_*α*_ is a real number vector with dimension *d*_attn_, and *W*_*α*_ is a real number matrix with shape (*d*_attn_, *d*_*h*_). From *i*=1 to len, the algorithm iterates as follows:

(2) $$\begin{array}{c} {\tilde{\alpha }}_{i}\;=\;v_{\alpha }^{T}\cdot \;\tanh\left(W_{\alpha }\cdot h_{i}\right), \end{array}$$

where *v*_*α*_^*T*^ is the transpose of *v*_*α*_.

For normalization that Sum({*α*_*i*_*|*1 ≤ *i* ≤ len})=1, softmax function is called to generate *α*_*i*_, i.e., $\left\{\alpha_{i}|1\leq i\leq \text{len}\right\}=\text{Softmax}\left(\left\{{\tilde{\alpha }}_{i}|1\leq i\leq \text{len}\right\}\right)$. From *i*=1 to len, the algorithm iterates as follows:

(3) $$\begin{array}{c} \alpha_{i}\;=\frac{\exp\left({\overset{˜}{\alpha }}_{i}\;\right)}{\sum_{j=1}^{\text{len}}\exp\left({\overset{˜}{\alpha }}_{j}\right)}, \end{array}$$

where exp(. ) is the exponential function.

We take a weighted sum of all states {*h*_*i*_*|*1 ≤ *i* ≤ len} as computing an expected state *h*_sum_ with dimension *d*_*h*_, which is similar to *h*_len_. The formula reads as follows:

(4) $$\begin{array}{c} h_{\text{sum}}=\sum_{i=1}^{\text{len}}\alpha_{i}h_{i}. \end{array}$$

Weighted sum state *h*_sum_ is computed to generate real number output *y* via fully connected dense layer whose activation is logistic sigmoid function.

### 3.3. Multi-Head Attention

In 2017, Vaswani et al. [4] introduced the multi-head attention mechanism, which consists of several attention heads running in parallel. Then, they built the Transformer without any recurrence or convolution to improve the machine translation quality. In addition, the Transformer is more parallelizable so as to require significantly less time to train.

In this paper, we propose a simplified Transformer, called Mini-Transformer, for the classification of news headline text data. Mini-Transformer is composed of multi-head attention layers and eschews recurrence or convolution.

For single-head dot-product attention, given an input sequence *X*={*x*_1_, *x*_2_, *x*_3_,…, *x*_len_} with length len, where {*x*_*t*_*|*1 ≤ *t* ≤ len} are real number vectors with dimension *d*_*x*_, we generate *Q* (Query), *K* (Key), and *V* (Value) with trainable parameter matrices, *W*_*q*_, *W*_*k*_, and *W*_*v*_. They are all real number matrices with shape (*d*_head_, *d*_*x*_), where *d*_head_ denotes dimension of attention head. The formulas are as follows:

(5) $$\begin{array}{c} q_{i}=W_{q}\cdot x_{i},Q=\left\{q_{i}|\;1\leq i\leq \text{len}\right\}=\text{query}\left(W_{q},X\right),\\ k_{i}=W_{k}\cdot x_{i},K=\left\{k_{i}|\;1\leq i\leq \text{len}\right\}=\text{key}\left(W_{k},X\right),\\ v_{i}=W_{v}\cdot x_{i},V=\left\{v_{i}|\;1\leq i\leq \text{len}\right\}=\text{value}\left(W_{v},X\right). \end{array}$$

After generating *Q*, *K*, and *V* with shape (len, *d*_head_), we compute single dot-product attention head as follows:

(6) $$\begin{array}{c} \text{head}=\text{Attention}\left(Q,K,V\right)=\text{Softmax}\left(Q\cdot K^{T}\right)\cdot V. \end{array}$$

The above dot-product single-head attention outputs a real number matrix with shape (len, *d*_head_). For multi-head attention, we employ *n*_head_ parallel attention heads. Due to the reduced dimension of each head (*d*_head_), the total computational cost is about the same as that of single-head attention with full dimensionality, but multi-head attention is more parallelizable for GPU to train. The formulas are as follows:

(7) $$\begin{array}{c} {\text{head}}_{j}=\text{Attention}\left(Q_{j},K_{j},V_{j}\right)\\ =\text{Softmax}\left(Q_{j}\cdot K_{j}^{T}\right)\cdot V_{j}\\ =\text{Softmax}\left(\text{query}\left(W_{qj},X\right)\cdot {\left(\text{key}\left(W_{kj},X\right)\right)}^{T}\right)\cdot \text{value}\left(W_{vj},X\right),\\ \text{multi-head}=\text{Concat}\left(\left\{{\text{head}}_{j}|1\leq j\leq n_{\text{head}}\right\}\right). \end{array}$$

**Figure 2:**
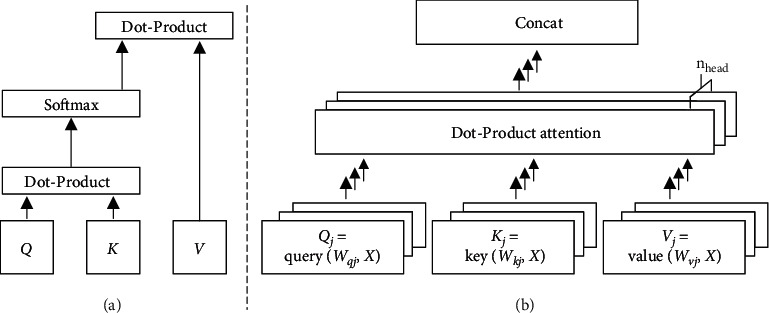
(a) Dot-Product attention. (b) Multi-Head attention consists of parallel attention heads.

Finally, multi-head attention outputs a real number matrix with shape (len, *n*_head_ × *d*_head_), as depicted in Figure 2.

## 4. Fraudulent News Headline Detection

Our work focuses on classifying massive news headlines data into fraudulent class (label 1) and true class (label 0). There are three Deep Learning models based on LSTM, LSTM with attention layer, and Mini-Transformer, respectively. The proposed scheme consists of labeled data source, text data preprocessing and training, and test and evaluation of Deep Learning models.

### 4.1. Scheme Flow Chart

**Figure 3:**
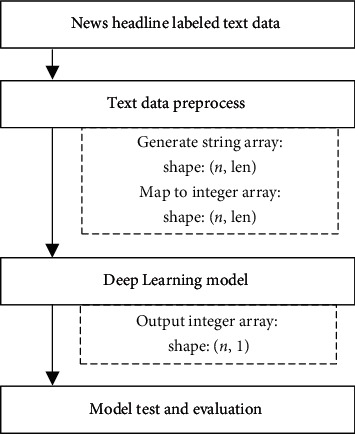
Fraudulent news headline detection scheme.

The flow chart of our proposed scheme for fraudulent news headline detection is shown in Figure 3.

### 4.2. Labeled Dataset

Three data sources are used in this paper. All of them are publicly available at Kaggle, the world's largest data science community [9].

If the length of a news headline is greater than or equal to 7, the news headline would be considered as valid news headline data. For balanced sampling, there are a total of 1,481,814 news headlines, including 736,009 items with label 1 and 745,805 items with label 0.

Fraudulent news headline dataset is The Examiner - Spam Clickbait Catalog [10]. Original source is the pseudo news site, The Examiner. At a certain point, the site was the 10th largest site on mobile and was attracting twenty million unique visitors per month. However, The Examiner no longer exists at present, Kaggle keeps the last record. Our work focuses on the fraudulent news headlines from January 1, 2013, to December 31, 2015, a total of 736,009 fraudulent news headlines (with a class label of 1).

True news headline datasets are A Million News Headlines [11] and News Category Dataset [12], a total of 745,805 true news headlines (with a class label of 0).

For A Million News Headlines, the original source is Australian Broadcasting Corporation. It includes the entire corpus of articles published by the ABC news website. With a volume of two hundred articles per day and a good focus on international news, every event of significance has been captured. It contains a total of 577,264 true news headlines from February 19, 2003, to December 31, 2019.

For News Category Dataset, the original source is HuffPost. Each news headline has a corresponding category (e.g., parenting, style and beauty, entertainment, wellness, and politics). It contains a total of 168,541 true news headlines from January 28, 2012, to May 26, 2018.

### 4.3. Text Data Preprocessing

We preprocess the original labeled news headline text data, including deleting repeated news headlines, removing unnecessary English symbols (i.e., ( ) ' ” , . ?: - ! #), removing redundant space characters, NLTK Lemmatization [13], truncating the news headlines that are too long, padding the news headlines that are too short, and converting uppercase letters to lowercase, etc.

The data with time order is generally called sequence. In this paper, news headline text data are typical sequences. The representation of news headlines is a two-dimensional string array with shape (*n*, len), where *n*=1,481,814 is the total number of news headlines, and len is the maximum length of news headlines. For example, the two-dimensional string array can be as follows:

- [‘tom', ‘act', ‘*a*', ‘cat', ‘*o*',…] 0
- [‘jerry', ‘act', ‘*a*', ‘mouse', ‘*e*',…] 0
- [‘goofy', ‘act', ‘the' , ‘sanguine', ‘dog',…] 0
- [‘jerry', ‘act', ‘the' , ‘hypothetical', ‘cat',…] 1,

where label 0 denotes true class and label 1 denotes fraudulent class.

We calculate the frequency of each English word in the two-dimensional string array, so as to identify high-frequency words and generate a high-frequency word dictionary. Significantly, in the procedure of generating the high-frequency word dictionary, our work mainly focuses on ignoring the extremely short words, marking stopwords [14] uniformly with tag 1 and marking low-frequency words uniformly with tag 2. For example, the word dictionary can be as follows:

- Stopword: ‘*a*' ->1, ‘the' ->1,…
- Low-Frequency word: ‘sanguine' ->2, ‘hypothetical' ->2,…
- High-Frequency word: ‘act' ->3, ‘cat' ->4, ‘jerry' ->5, ‘dog' ->6, ‘goofy' ->7, ‘mouse'->8, ‘tom' ->9,…,

where ‘*a*' and ‘the' are stopwords marked uniformly with tag 1, ‘sanguine' and ‘hypothetical' are low-frequency words marked uniformly with tag 2.

The original news headline is composed of several words; to facilitate the training and test of Deep Learning model, we map each word string to the corresponding integer based on the generated word dictionary, thus the news headline two-dimensional string array can be converted to a two-dimensional integer array with shape (*n*, len), e.g., the two-dimensional integer array can be as follows:

- [9, 3, 1, 4,…] 0
- [5, 3, 1, 8,…] 0
- [7, 3, 1, 2, 6,…] 0
- [5, 3, 1, 2, 4,…] 1,

where ‘o' and ‘e' are extremely short and unnecessary words (only one letter) that have been ignored, tag 1 denotes all stopwords, tag 2 denotes all low-frequency words, and tags which are greater than or equal to 3 denote corresponding high-frequency words.

### 4.4. Deep Learning Model Structure

**Figure 4:**
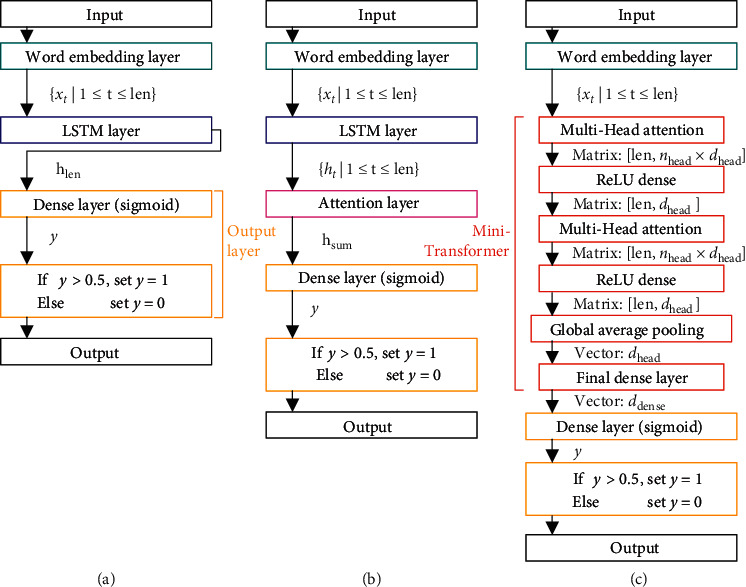
(a) LSTM. (b) LSTM with attention layer. (c) Mini-Transformer.

In this section, we propose three Deep Learning models, all of them contain word embedding layers and output layers; their structures are shown in Figure 4.

Word2Vec [15] provides a simple and effective method for vectorized representation of words, which can be employed in word embedding task. In word embedding layer, each integer in news headline two-dimensional integer array will be converted to real number vector with dimension *d*_*x*_. Eventually, the two-dimensional integer array will be converted to a real number array with shape (*n*, len, *d*_*x*_), i.e., each piece of news headline text data will be converted to word vector input sequence *X*={*x*_1_, *x*_2_, *x*_3_,…, *x*_len_}.

In output layer, vector *h*_len_, vector *h*_sum_, or the vector with dimension *d*_dense_ returned from final dense layer in Mini-Transformer will be converted to real number *y* via dense layer whose activation is logistic sigmoid function. If *y* is greater than threshold 0.5, it will be set to 1, else it will be set to 0.

In Mini-Transformer, we employ two layers of multi-head attention sublayer and fully connected dense sublayer without bias. It is worth noting that the activation function of dense sublayer is Rectified Linear Unit [16] (ReLU), but final dense layer has no activation function.

## 5. Experimental Settings and Results

If the frequency of a word is greater than or equal to 140 times, the word will be considered as high-frequency word; from word frequency statistics, the total number of high-frequency words is 7,996, so the length of word dictionary is 7,998, including low-frequency words and stopwords.

To configure the Deep Learning model for training, in tf.keras.Model.compile, we set that optimizer = Adam(learning_rate = 0.0002), loss = BinaryCrossentropy().

For word embedding layer, dimension of word vectors {*x*_*t*_*|*1 ≤ *t* ≤ len}(*d*_*x*_) is 25 and the maximum length of news headlines (len) is 15. For LSTM and attention layer, dimension of hidden states {*h*_*t*_*|*1 ≤ *t* ≤ len}(*d*_*h*_) is 16 and *d*_attn_ is 32.

In Mini-Transformer, for multi-head attention sublayer, number of dot-product attention heads (*n*_head_) is 8 and dimension of that (*d*_head_) is 64, and for final dense layer, *d*_dense_ is 16, which is the same as *d*_*h*_.

After shuffled, 80% of original labeled dataset is split into the training set and 20% is split into the test set for cross-validation; for batch training [17], we combine consecutive news headlines of this text dataset into batches, batch size is set to 768.

**Figure 5:**
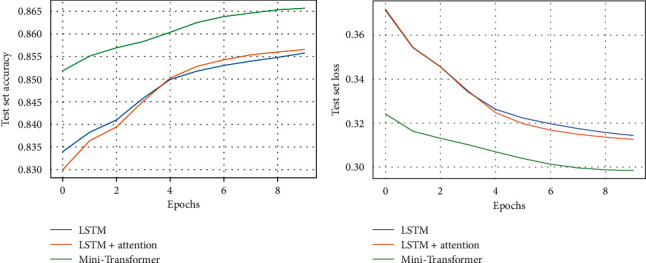
(a) Test set accuracy. (b) Test set loss.

For LSTM, LSTM with attention layer and Mini-Transformer, test set accuracy and loss curves in 10 epochs are shown in Figure 5; accuracy, precision, recall, and F1 score are shown in Table 1.

For further comparison with the models from [18], we conducted several contrast experiments by employing three regular Machine Learning models: logistic regression [19], linear support vector machine (Linear SVM) [20], and random forest [21].

For logistic regression, primal formulation is implemented with liblinear solver (dual = false). For Linear SVM, the algorithm is selected to solve primal optimization problem (dual = false). For random forest, the minimum number of samples required to split an internal node is 50 (min_samples_split = 50). Other hyper-parameters are default from scikit-learn.

From Table 1, three regular Machine Learning models do not achieve good classification results, this may be because they are too simplistic to process massive news headline data.

Compared with LSTM, Mini-Transformer achieves an obvious accuracy improvement in classification performance (0.9%–1.0%). However, LSTM with attention layer achieves a slight accuracy improvement in classification performance (<0.1%); this may be because the maximum length of news headlines (len) is so short that general attention layer cannot play a sufficient role in reflecting the importance of all hidden states returned from LSTM layer.

## 6. Conclusion

Existing work has not focused on fraudulent news headline detection. In this paper, we have compared the classification performance of mainstream LSTM network and general attention mechanism for fraudulent news headline detection using massive news headline data, which is helpful for the research on the defense system against social engineering attacks.

In addition, according to relevant experience, we have built a more advanced Deep Learning model, Mini-Transformer, which further improves the classification performance.

There is still room to optimize the proposed Deep Learning model. For future work, we can employ Bidirectional Encoder Representations from Transformers (BERT) as NLP pre-training method. Additionally, adversarial training and virtual adversarial training may be beneficial to improving the classification performance.

## Figures and Tables

**Table 1:** Accuracy, precision, recall, and F1 score.

Model	Accuracy (%)	Class	Precision (%)	Recall (%)	F1 score
Logistic regression	63.2167	Fraudulent	69.7941	45.7810	0.5529
		True	60.0367	80.4344	0.6875

Linear SVM	62.7882	Fraudulent	69.5541	44.6495	0.5439
		True	59.6196	80.7000	0.6858

Random forest	71.6277	Fraudulent	71.2205	71.9842	0.7160
		True	72.0385	71.2757	0.7166

LSTM	85.5761	Fraudulent	83.7513	88.0529	0.8585
		True	87.5720	83.1304	0.8529

LSTM + attention	85.6551	Fraudulent	83.8343	88.1208	0.8592
		True	87.6456	83.2202	0.8538
Mini-Transformer	86.5692	Fraudulent	84.6380	89.1490	0.8683
		True	88.6894	84.0216	0.8629

## Data Availability

The data used to support the findings in this study are available from The Examiner-Spam Clickbait Catalog|Kaggle: https://www.kaggle.com/therohk/examine-the-examiner, A Million News Headlines|Kaggle: https://www.kaggle.com/therohk/million-headlines, and News Category Dataset|Kaggle: https://www.kaggle.com/rmisra/news-category-dataset.

## References

[1] *Symantec Internet Security Threat Report*.

[2] Hochreiter S., Schmidhuber J. (1997). Long short-term memory. *Neural Computation*.

[3] Bahdanau D., Cho K., Bengio Y. Neural machine translation by jointly learning to align and translate.

[4] Vaswani A., Shazeer N., Parmar N. Attention is all you need.

[5] Castillo C., Mendoza M., Poblete B. Information credibility on twitter.

[6] Ma J., Gao W., Mitra P. Detecting rumors from microblogs with recurrent neural networks.

[7] Guo H., Cao J., Zhang Y., Guo J., Li J. Rumor detection with hierarchical social attention network.

[8] Song M., Park H., Shin K.-S. (2019). Attention-based long short-term memory network using sentiment lexicon embedding for aspect-level sentiment analysis in Korean. *Information Processing & Management*.

[9] Kaggle https://www.kaggle.com.

[10] The Examiner - Spam Clickbait Catalog | Kaggle, 6 Years of Crowd Sourced Journalism, Rohit Kulkarni, https://www.kaggle.com/therohk/examine-the-examiner

[11] *A Million News Headlines | Kaggle*.

[12] *News Category Dataset | Kaggle*.

[13] Bird S., Klein E., Loper E. (2009). *Natural Language Processing with Python: Analyzing Text with the Natural Language Toolkit*.

[14] *Default English stopwords list from Ranks NL Webmaster Tools*.

[15] Mikolov T., Sutskever I., Chen K., Corrado G., Dean J. Distributed representations of words and phrases and their compositionality.

[16] Glorot X., Bordes A., Bengio Y. Deep sparse rectifier neural networks.

[17] Keskar N. S., Mudigere D., Nocedal J., Smelyanskiy M., Tang P. T. P. On large-batch training for deep learning: generalization gap and sharp minima.

[18] Rajesh K., Kumar A., Kadu R. Fraudulent news detection using machine learning approaches.

[19] Cramer J. S. (2002). *The Origins of Logistic Regression*.

[20] Bradley P. S., Mangasarian O. L. (2000). Massive data discrimination via linear support vector machines. *Optimization Methods and Software*.

[21] Breiman L. (2001). Random forests. *Machine Learning*.

